# A Scalable Quality Assurance Process for Curating Oncology Electronic Health Records: The Project GENIE Biopharma Collaborative Approach

**DOI:** 10.1200/CCI.21.00105

**Published:** 2022-02-22

**Authors:** Jessica A. Lavery, Eva M. Lepisto, Samantha Brown, Hira Rizvi, Caroline McCarthy, Michele LeNoue-Newton, Celeste Yu, Jasme Lee, Xindi Guo, Thomas Yu, Julia Rudolph, Shawn Sweeney, Ben Ho Park, Jeremy L. Warner, Philippe L. Bedard, Gregory Riely, Deborah Schrag, Katherine S. Panageas

**Affiliations:** ^1^ Memorial Sloan Kettering Cancer Center, New York, NY; ^2^ Division of Population Sciences, Dana-Farber Cancer Institute Boston, MA; ^3^ Vanderbilt Ingram Cancer Center, Nashville, TN; ^4^ Princess Margaret Cancer Centre, University Health Network, Toronto, ON; ^5^ Sage Bionetworks, Seattle, WA; ^6^ American Association for Cancer Research, Philadelphia, PA

## Abstract

The American Association for Cancer Research Project Genomics Evidence Neoplasia Information Exchange Biopharma Collaborative is a multi-institution effort to build a pan-cancer repository of genomic and clinical data curated from the electronic health record. For the research community to be confident that data extracted from electronic health record text are reliable, transparency of the approach used to ensure data quality is essential.

**MATERIALS AND METHODS:**

Four institutions participating in AACR's Project GENIE created an observational cohort of patients with cancer for whom tumor molecular profiling data, therapeutic exposures, and treatment outcomes are available and will be shared publicly with the research community. A comprehensive approach to quality assurance included assessments of (1) feasibility of the curation model through pressure test cases; (2) accuracy through programmatic queries and comparison with source data; and (3) reproducibility via double curation and code review.

**RESULTS:**

Assessments of feasibility resulted in critical modifications to the curation directives. Queries and comparison with source data identified errors that were rectified via data correction and curator retraining. Assessment of intercurator reliability indicated a reliable curation model.

**CONCLUSION:**

The transparent quality assurance processes for the GENIE BPC data ensure that the data can be used for analyses that support clinical decision making and advances in precision oncology.

## INTRODUCTION

The future of precision medicine in oncology requires detailed patient data alongside molecular characterization of tumors to allow for discovery, risk stratification and ultimately, to inform the selection of optimal therapy.^1^ Although efforts such as The Cancer Genome Atlas have molecularly characterized more than 200,000 primary cancer tumors and have led to insights regarding the genomic landscape of many cancers, phenomic (clinical) data that includes clinical characteristics, therapeutic exposures, and salient outcomes are limited in The Cancer Genome Atlas and similar data sources.^2^ Historically, with limited treatment options and short survival for patients with advanced cancer, overall survival (OS) was viewed as the most relevant end point for clinical decision making. With more treatments now available and the need to make more rapid treatment decisions, intermediate end points such as progression-free survival (PFS) are routinely used for patients treated as part of clinical trials. However, with the majority of patient care occurring outside of the clinical trial setting, there is a need for robust curation of intermediate end points such as real-world progression-free survival. Much of the critical data characterizing end points such as treatment duration, toxicity, progression, and recurrence are not captured using structured data fields in the electronic health record (EHR), posing challenges for the collection and synthesis of these data.^3^

**CONTEXT**

**Key Objective**
To develop a scalable quality assurance process for a multi-institution initiative to curate disease characteristics, treatment, and outcomes from the electronic health record for patients with cancer.
**Knowledge Generated**
The transparent quality assurance processes and their findings ensure that the GENIE BPC data can be used for analyses that support clinical decision making and advances in precision oncology.
**Relevance**
These processes may also be adapted for other large-scale curation initiatives.

The American Association for Cancer Research (AACR) Project Genomics Evidence Neoplasia Information Exchange Biopharma Collaborative (GENIE BPC) is a multi-institution effort to build a pan-cancer data repository of genomic, therapeutic, and phenomic data curated from the EHR. The GENIE BPC project builds on AACR Project GENIE, a publicly available registry of genomic data and limited clinical data from 19 cancer centers internationally.^4-6^ To accomplish the goals of improving clinical decision making on the basis of real-world data, the existing Project GENIE genomic data in conjunction with the PRISSMM framework^7,8^ to curate phenomic information from the EHR were leveraged to create a comprehensive data set characterizing the cancer treatment trajectories and outcomes of approximately 7,500 patients with cancer with six distinct cancer types.

The utility of the scientific research stemming from this open-access data source depends on having high-quality curated data that have undergone rigorous quality assurance (QA) processes to verify the completeness and accuracy of essential data elements. Even if performed at a single institution for a single cancer type, EHR data curation is complex and time-consuming; if not performed with appropriate quality control measures, the resulting data will be limited in its usability and generalizability. Harmonizing data curation is especially challenging when projects span multiple institutions that rely on distinct EHRs and informatics ecosystems. Furthermore, there are differences in provider care patterns and note structure, requiring coordination between institutions to ensure that data are consistently collected. For the research community to be confident that the data extracted from the EHR are reliable and reproducible, transparency of the QA approach is essential. This manuscript provides an overview of the GENIE BPC project and the approach used to ensure quality, rigor, and reproducibility so that the resulting data can support analyses that advance the goals of precision medicine.

## MATERIALS AND METHODS

The AACR Project GENIE consortium has aggregated data from more than 120,000 tumors that have undergone high-throughput sequencing using targeted gene panels. Data from the sequencing reports and select clinical data elements, most notably age and OS, are publicly available, and have been previously described.^9^ The focus of the GENIE BPC project is to augment the genomic information with detailed phenomic data from the EHR and other institutional sources, such as the tumor registry, that characterize baseline patient and tumor attributes, treatment exposures, and clinically relevant outcomes.

The initial phase of the GENIE BPC project included curation of six cancer cohorts: non–small-cell lung cancer (NSCLC; N = 1876), colorectal cancer (CRC; N = 1,501), breast cancer (N = 1,130), pancreatic cancer (N = 1,125), prostate cancer (N = 1,125), and bladder cancer (N = 750). Cases were randomly selected for phenomic data curation if the patient was age ≥ 18 years at the time of genomic sequencing, the sample met inclusion criteria (stage at diagnosis; eligible OncoTree code) and was performed during a specified time period, and eligible for 2 years of follow-up after sequencing. Each cancer-specific cohort is curated using the same data model with minor adaptations on the basis of features that are specific to the cancer type, such as human epidermal growth factor receptor 2 status for breast cancer. Data are curated locally at each institution and uploaded to a centralized data repository. There are four participating institutions: Memorial Sloan Kettering Cancer Center (MSKCC), Dana Farber Cancer Institute, Vanderbilt Ingram Cancer Center, and the University Health Network Princess Margaret Cancer Centre.

The phenomic, therapeutic, and oncologic outcome data are curated from the EHR and other institutional data sources according to the patient- and cancer-centric PRISSMM framework for determination of real-world outcomes. PRISSMM uses a cancer-agnostic curation model that standardizes key components of outcome ascertainment in oncology on the basis of existing data standards. The PRISSMM framework for defining cancer outcomes includes data collection with respect to pathology; radiology; imaging; signs and symptoms; tumor markers; and medical oncologist assessments.^7,8,10^ Specifically, all pathology reports, all radiology reports for imaging studies other than plain x-rays and ultrasounds, and one medical oncologist note per month are curated. Curation is partially automated; select variables (< 10%) that are recorded in the institution's tumor registry are transferred directly into the GENIE BPC database, whereas other variables are manually extracted from the EHR. Curated data are stored in a Research Electronic Data Capture (REDCap) database of more than 700 data fields structured with a REDCap instrument corresponding to each of the PRISSMM modules. Although the data dictionary is uniform across institutions, different EHR systems and versions of REDCap data management software across institutions add a layer of complexity to ensuring consistent quality.

### Project Teams

Phenomic data curation across institutions requires a dedicated team at each institution with expertise in both informatics and clinical oncology, centralized project coordination and management, a centralized data repository and host, and a governance structure to facilitate streamlined decision making. The AACR Project GENIE Coordinating Center oversees management of the project and liaises between the biopharmaceutical partners and participating institutions that contribute data. Sage Bionetworks maintains the centralized data repository and is responsible for assuring data deidentification, versioning, provenance, access, and release internally and publicly. Each participating institution has a team of curators who abstract data from the medical record and QA manager(s) who are experts in clinical oncology research and responsible for training curators and implementing and overseeing the QA processes. The Statistical Coordinating Center (SCC) is centralized at MSKCC and is responsible for supporting QA processes, providing feedback to institutions, stipulating the need for reabstraction of fields with unacceptably high rates of discordance, and preparing the final comprehensive data set for release, including the derivation of variables and their respective documentation.

### QA Processes

**FIG 1.**
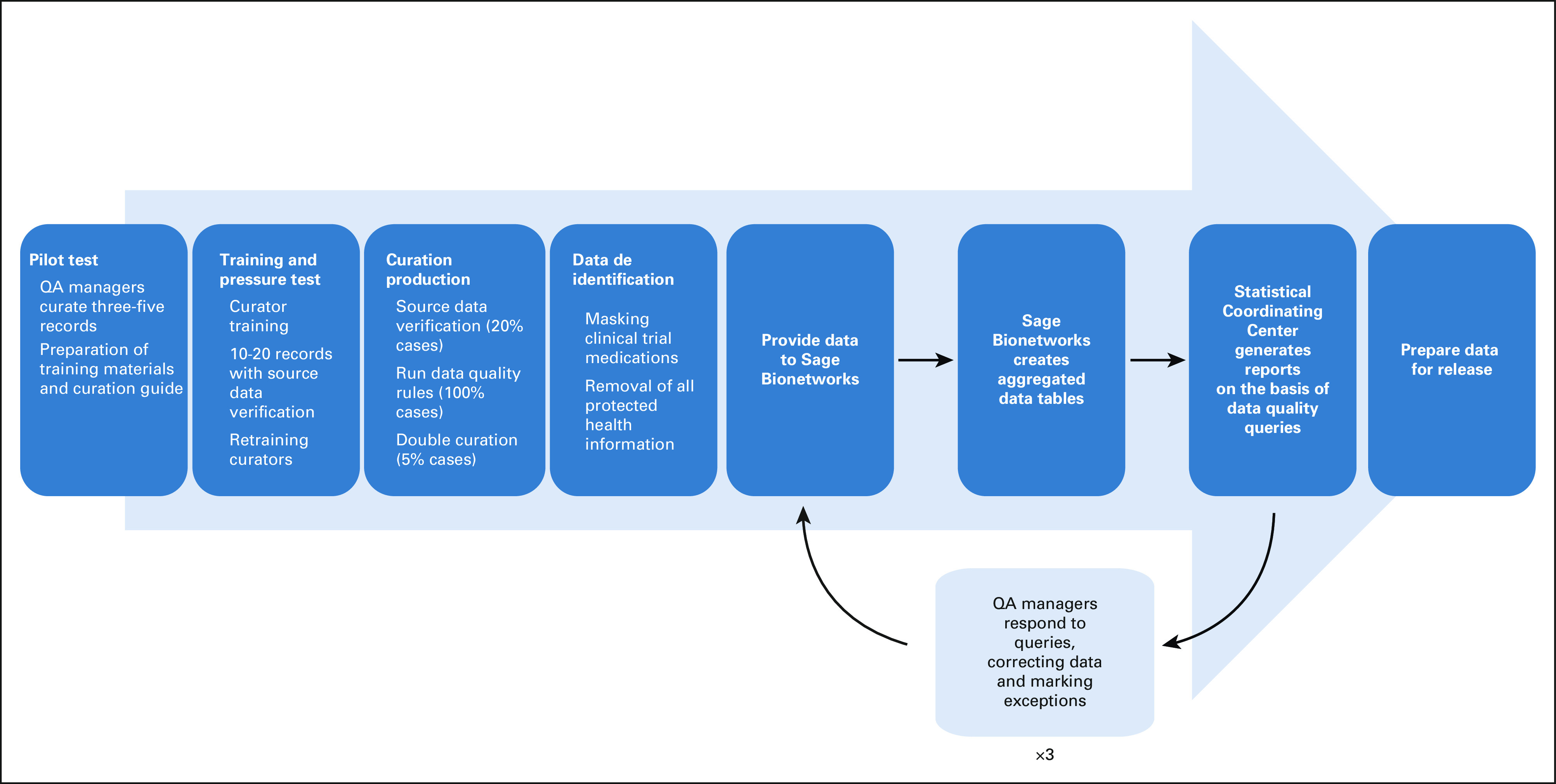
Flow diagram for QA processes and data release. QA, quality assurance.

Curation initiatives require assessment of different components of data quality throughout the curation process. Multipronged QA processes were developed at the start of the project to address feasibility, accuracy, and reproducibility (Table 1; Fig 1). We describe each process and its role in ensuring the quality of the data. At the time of this writing, the NSCLC, CRC, and breast cancer data have been curated; summaries of the results of the QA processes for these cancer sites are presented.

**TABLE 1.**
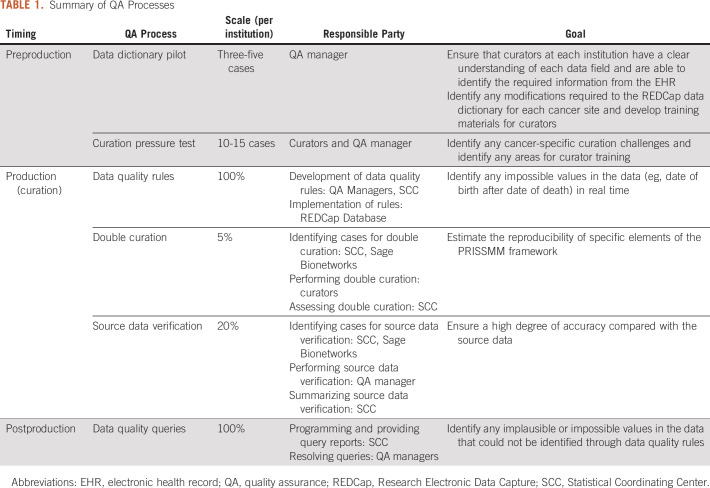
Summary of QA Processes

#### Assessing feasibility of the curation model.

At the initiation of curation of every new cancer cohort, a pilot phase is conducted to assess the feasibility of the curation model. Although PRISSMM is a pan-cancer curation model, there are distinctions in the recording of data in the EHR across cancer sites. QA managers curate between three and five cases to assess the curation directives for each new cancer site. The goal of the pilot curation phase is to ensure that the data directives are comprehensible, cancer-specific modifications can be operationalized, and training materials for curators cover both common and ambiguous cases with specific examples. During this phase, QA managers from participating institutions contribute feedback on the content of the database, the training directives, and the feasibility of extracting specific elements in their local environments. Modifications are made to the REDCap data dictionary, the accompanying curation guide, and the training materials, as needed.

After the pilot phase, curator training is performed by QA managers using the training directives with cancer-specific data elements and directives, which indicate the documents in the EHR that are in scope for review and abstraction under the PRISSMM framework. Curators then review examples and a test case with QA managers. This process ensures that curators are abstracting relevant information from the EHR consistently.

Following curator training, between 10 and 15 cases (one-two cases per curator) are curated at each institution, which are then fully reviewed by the QA Manager to ensure accuracy against the source data as part of a curation pressure test. The QA manager systematically records discrepancies. On the basis of the results of this source data verification, the data elements and/or directives may be modified; if data elements or directives are updated, curators at all institutions are retrained before production (Fig 1). If substantial modifications are required, then a second set of pressure test cases may be curated.

#### Assessing curated data accuracy.

**FIG A1.**
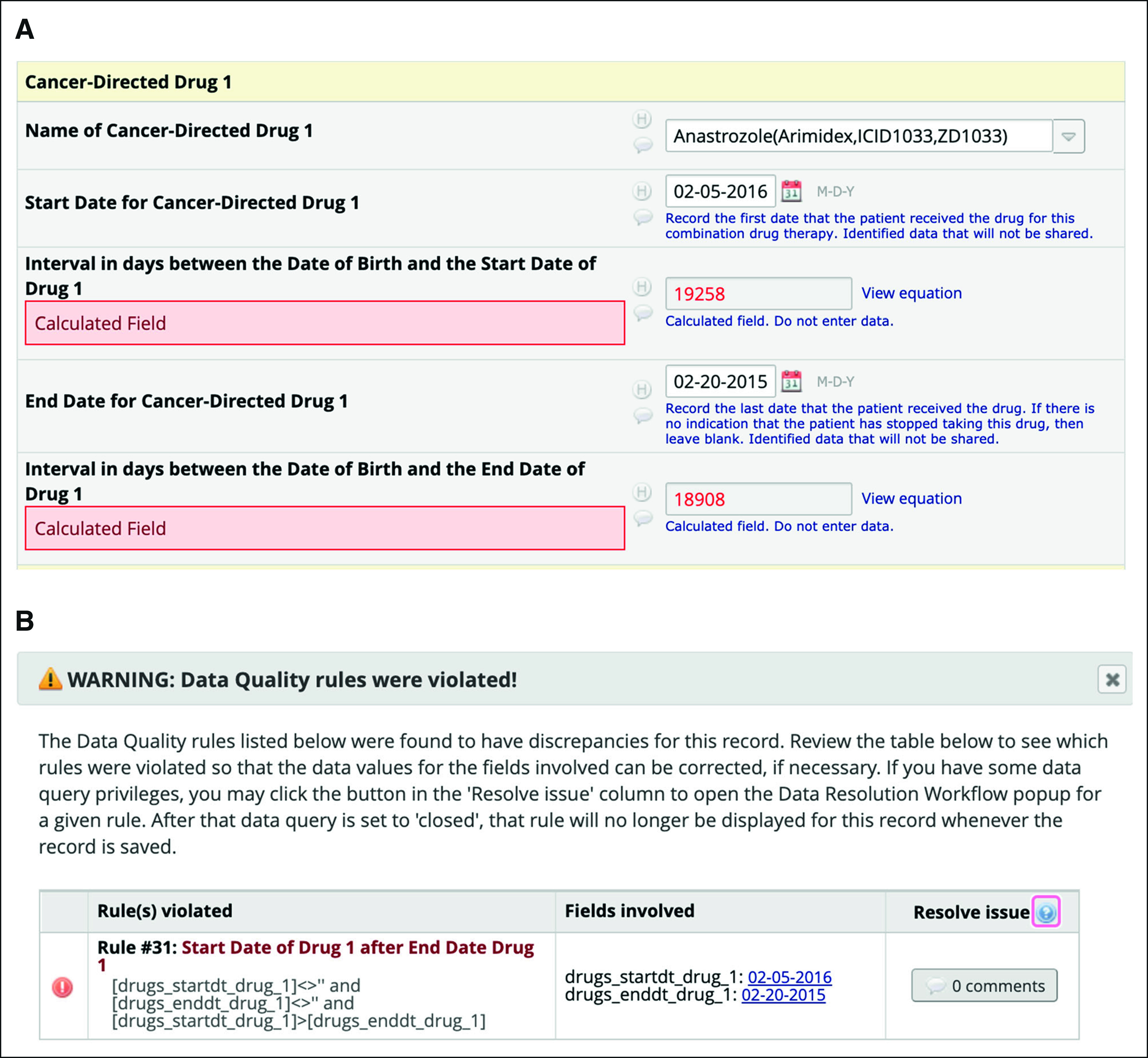
Example of (A) incorrectly curated data and (B) corresponding REDCap data quality rule alert. Example REDCap data quality rule for incorrectly curated data on the basis of simulated data for an example patient. REDCap, Research Electronic Data Capture.

Data quality rules refer to programmatic checks implemented in the REDCap project that are executed in real time during curation. The data quality rules aim to identify missing data and any impossible values, for example, a date of death preceding the date of birth. There are more than 150 data quality rules implemented as part of GENIE BPC (Table 2; Appendix Fig A1). Identification of inconsistencies and errors during curation allows for corrections to be implemented in real time. The number of records flagged by a rule and the number of exceptions recorded cannot currently be exported from REDCap and readily analyzed.

**TABLE 2.**
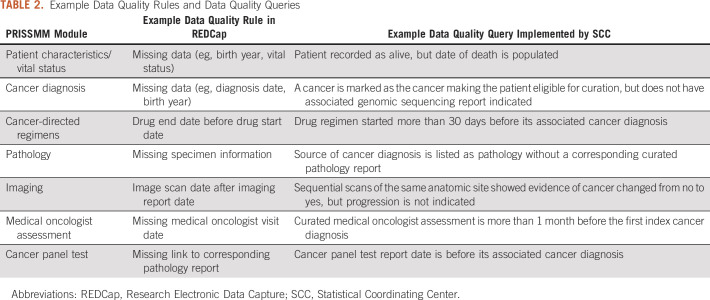
Example Data Quality Rules and Data Quality Queries

There are more than 75 data quality queries that address the consistency of data elements that cannot be compared using data quality rules because of REDCap limitations (Table 2). Data quality queries are implemented by the SCC after curation is complete on the data aggregated across institutions. For both data quality rules and queries, curators and QA managers determine whether the data require a correction; if not, the exception is documented. A minimum of three rounds of data quality queries are evaluated for each cancer cohort to ensure the data are of the highest integrity at the time of release.

**FIG A2.**
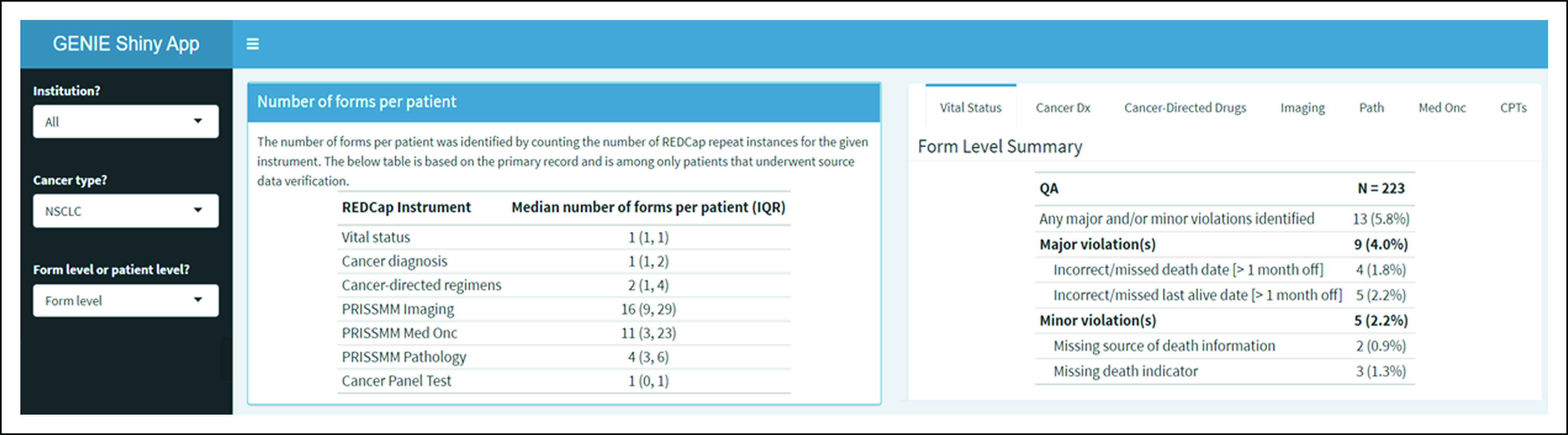
Snapshot of quality assurance application summarizing source data verification findings. The screenshot indicates a dropdown on the left-hand side where the user can specify a particular institution, cancer diagnosis, and summary level for the source data verification findings. The first table on the left shows the number of forms that were reviewed per patient. The table to the right has tabs for each PRISSMM module and shows the number of forms that were compared with the electronic health record and the extent and type of major and minor issues.

The accuracy of the curated data is also assessed through source data verification, a manual process requiring familiarity with the curation directives, clinical content, and the nuances of phenomic data documentation in the EHR. QA Managers at each institution perform source data verification on 20% of records that are randomly selected before beginning curation. Source data verification is performed after the case is fully curated, during production, so that any systematic issues identified across records can be rectified before completing curation of an entire cancer cohort. Discrepancies between the curated data and the EHR are classified into major, minor, and other issues (Table 3). The issues were categorized a priori by a team of clinical experts, taking into consideration the ramifications of erroneous data. Major issues are discrepancies that would affect data analysis of key outcome measures, such as incorrectly identified or missed progression on an imaging report. Minor issues, such as missing source of death information, represent discrepancies with lesser implications for analysis. Other issues refer to discrepancies that were not classified as major or minor, such as instances where the data are not incorrect but could be more specific. Although all discrepancies are corrected by QA managers, the classification into major and minor issues is used to systematically identify opportunities to revise curation directives and to initiate curator retraining. The SCC developed a QA application that updates automatically as data are centrally uploaded and summarizes the extent of issues within and across institutions (Appendix Fig A2). An acceptable benchmark regarding the number of violations is not easily defined; these data are regularly evaluated in a qualitative fashion to determine when and how to initiate corrections.

**TABLE 3.**
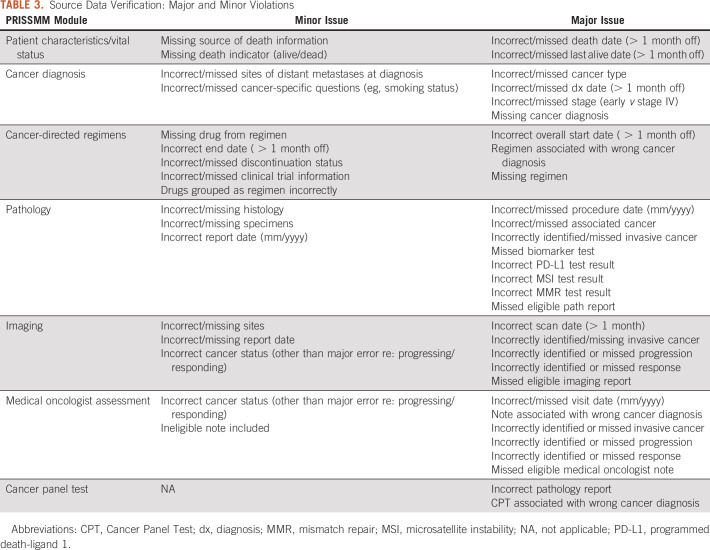
Source Data Verification: Major and Minor Violations

#### Assessing reproducibility.

Double curation was performed to evaluate intercurator reliability. For a predetermined randomly selected subset of 5% of cases at each institution, two curators independently abstracted data for the same patient. Agreement was assessed using the kappa statistic for categorical variables, concordance correlation coefficient for continuous variables, and a correlation statistic on the basis of iterative multiple imputation for time-to-event data.^11^

Statistical code review was performed by a second statistician to ensure reproducibility of the derivation of variables, including outcomes such as PFS and OS provided in the data releases. Any discrepancies were discussed within the SCC and resolved accordingly before data release.

## RESULTS

Over the course of curation, 34% (n = 647) NSCLC, 29% (n = 436) CRC, and 53% (n = 597) of patients with breast cancer had at least one data quality query. There were 1,074 queries identified for round one of NSCLC review, 404 for round two, and 65 for round three. For CRC, there were 699, 187, and 22 queries in rounds one, two, and three, respectively. For breast cancer, 846 queries were identified in round one, 350 in round two, and 154 in round three. Of the queries identified, 30% of NSCLC, 50% of CRC, and 49% of breast cancer queries did not result in a correction to the data (exception recorded). The high rates of queries designated as exceptions reflect an evolving process for evaluating data quality queries. If QA managers identify a common exception to a complex query, they provide feedback to the SCC so that the scenario is programmatically excluded from future query reports. Conversely, upon reviewing the data, the QA managers may recommend a new query be incorporated for subsequent rounds.

The findings of source data verification yielded similar patterns across the three cohorts. The percentage of forms with major issues ranged from < 1% (cancer panel test instrument) to 8% (imaging and medical oncologist instruments), and the percentage with minor violations ranged from < 1% (vital status and cancer panel test instruments) to 20% (cancer diagnosis instrument; Table 4). All inconsistencies were corrected and triggered partial review of records that were not selected for source data verification to ensure high quality for data fields found to have high error rates.

**TABLE 4.**
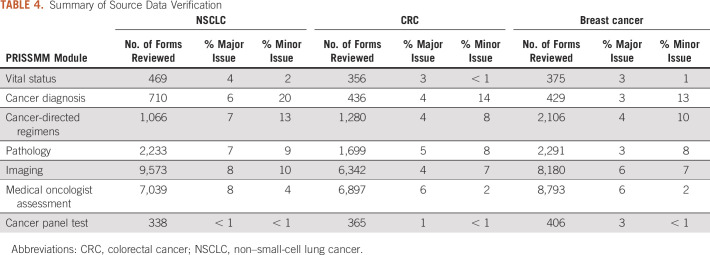
Summary of Source Data Verification

**FIG A3.**
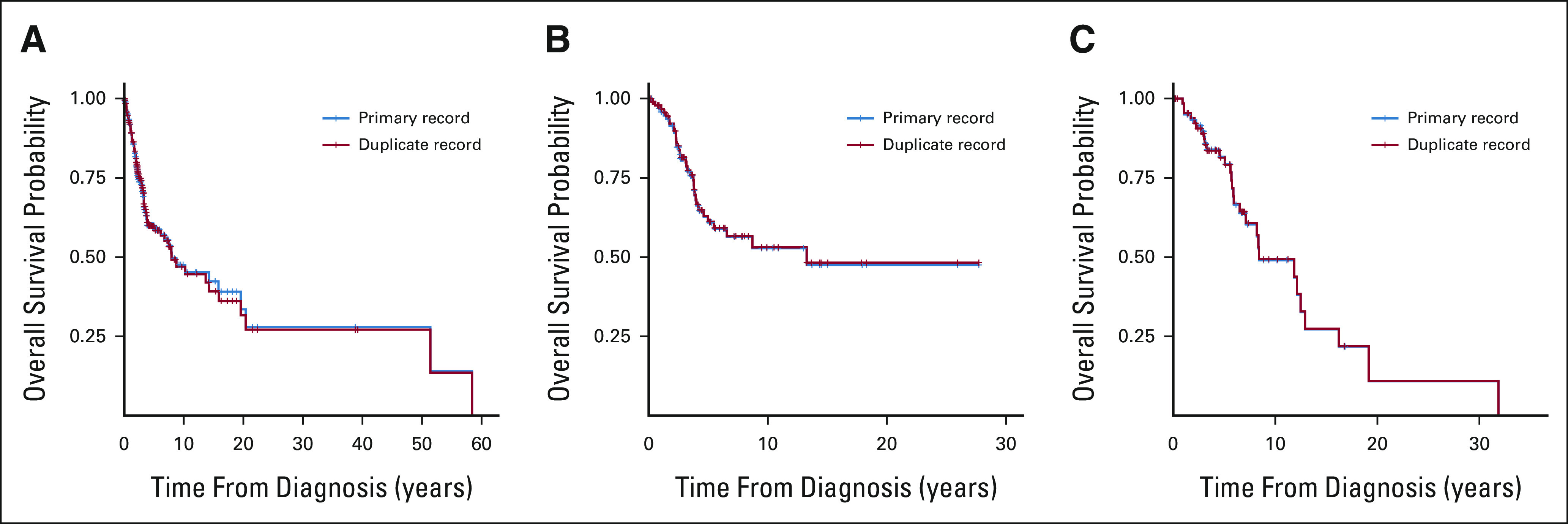
Kaplan-Meier estimates of OS stratified by curator for (A) NSCLC, (B) CRC, and (C) BrCa. Kaplan-Meier estimates of OS stratified by the primary curator and secondary curator among records that underwent double curation for the purposes of assessing reproducibility. Note that curves are not intended to describe estimates of time to event end points, but to demonstrate the assessment of reproducibility of curation of time to event end points across curators. BrCa, breast cancer; CRC, colorectal cancer; NSCLC, non–small-cell lung cancer; OS, overall survival.

**FIG A4.**
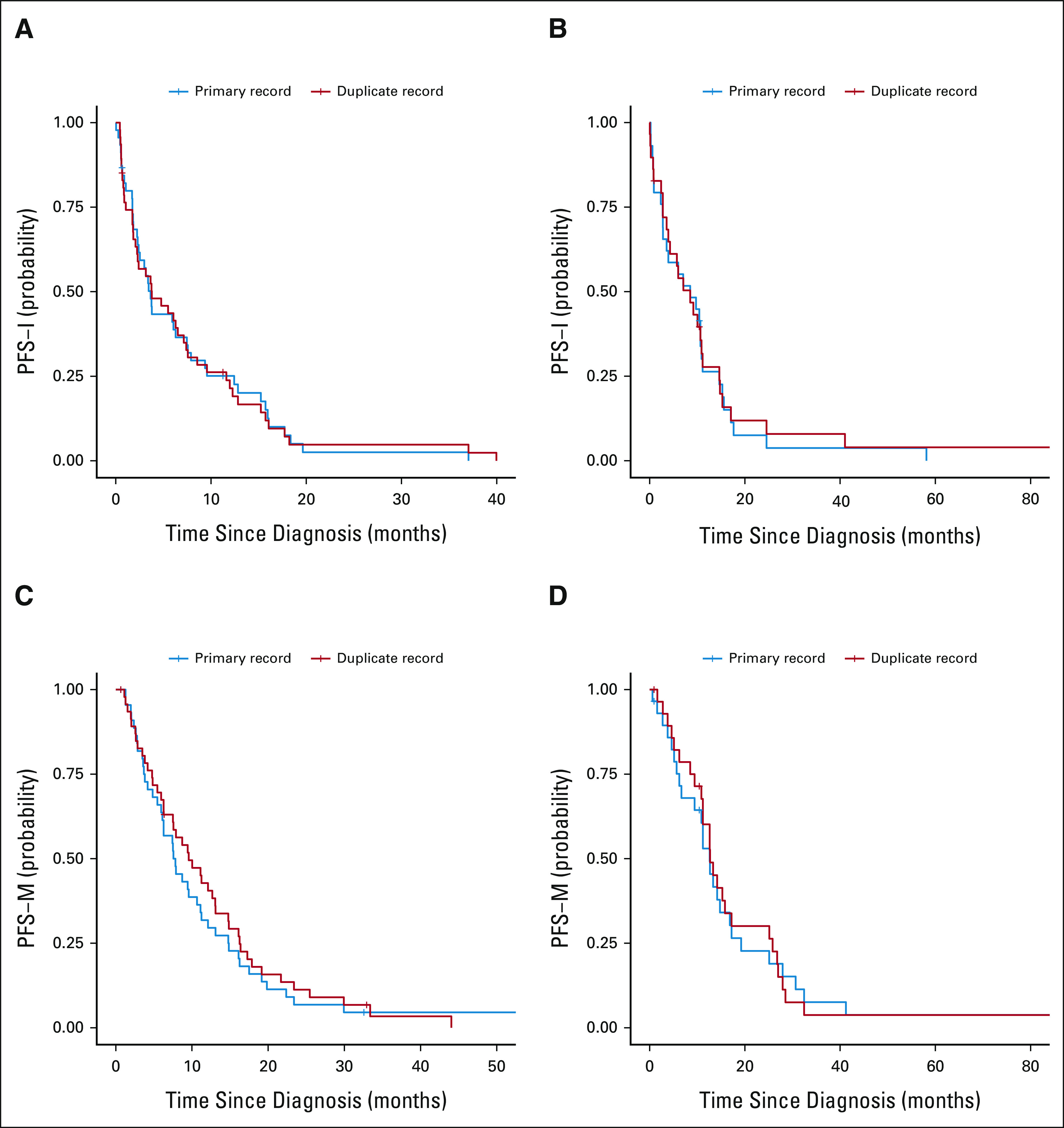
Kaplan-Meier estimates of PFS-I and PFS-M stratified by curator for NSCLC and CRC: (A) PFS-I NSCLC, (B) PFS-I CRC, (C) PFS-M NSCLC, and (D) PFS-M CRC. Survival curves are stratified by the primary curator and secondary curator among records that underwent double curation for the purposes of assessing reproducibility. Note that curves are not intended to describe estimates of time to event end points, but to demonstrate the assessment of reproducibility of curation of time to event end points across curators. CRC, colorectal cancer; NSCLC, non–small-cell lung cancer; PFS-I, progression-free survival according to imaging; PFS-M, progression-free survival according to medical oncologist.

Assessment of intercurator reliability for select variables on the cancer diagnosis, cancer-directed drug regimen, and cancer panel test instruments revealed adequate agreement between curators (kappa ≥ 0.6; concordance correlation coefficient > 0.6). Estimates of the survival distributions were similar when analyzing the double-curated cases (Table 5; Appendix Figs A3 and A4).

**TABLE 5.**
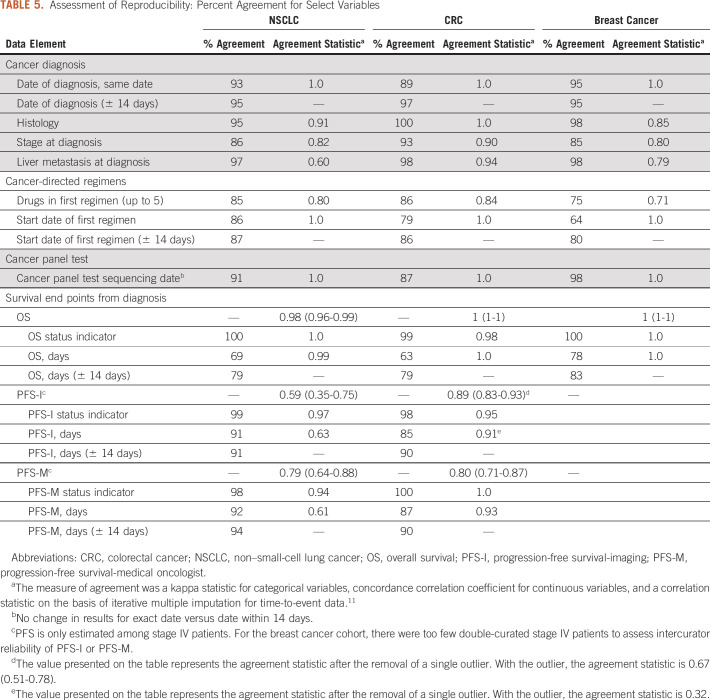
Assessment of Reproducibility: Percent Agreement for Select Variables

## DISCUSSION

The GENIE BPC Project represents a coordinated effort to generate a publicly released linked clinicogenomic data set that, when analyzed with methodologic rigor, can be used to further advance precision medicine in oncology. A comprehensive approach to QA was necessary to ensure high-quality data for curation of pan-cancer data from EHRs across multiple institutions. As initiatives to curate EHR data are increasingly used to structure data for research purposes and further may inform regulatory decisions, it is important to highlight the need for transparent QA processes that are uniform across institutions. To enhance rigor and reproducibility, such initiatives require the means to implement a QA process that addresses the feasibility, accuracy, and reproducibility of data curation. An extensible and portable QA process and built-in data checks facilitate the scalability of the project. A rigorous and scalable QA process for GENIE BPC was accomplished by a multipronged process targeting a subset of records for the most time-intensive QA processes (20% source data verification; 5% double curation), applying data quality rules and data quality queries to 100% of cases allowing for data corrections and the identification of systematic discrepancies.

Especially in a project of this scale, it is critical that QA processes involve harmonization of curation and QA achieved through frequent communication of project teams within and across institutions. The PRISSMM model was developed to standardize data collection across varied EHRs. Furthermore, the QA processes are performed on the harmonized data and are implemented uniformly. The QA processes described in this manuscript reflect a set of procedures that are optimal to this specific project in terms of balancing resources and data quality and have been refined based upon careful review of the curated data and a learning QA system. The QA processes use programmatic standardization and implementation when possible (uniform REDCap data dictionaries, data quality rules, and data quality queries), and rely on manual review for a subset of cases (source data verification) to systematically identify areas for improvement. Although the future of real-world data collection is advancing toward natural language processing for data abstraction, reducing the efforts and time required for human curation, rigorous QA processes that rely heavily on manual review would still be required to guarantee the accuracy of the data.^12^

Although the curation and QA processes can easily be extended to all solid tumors, adaptations to curator training, data abstraction, and QA methods may be required to maintain high-quality data for hematologic malignancies, which are distinct from solid tumors in terms of their characterization, treatment, and outcome ascertainment. For example, radiologic evidence of disease is a key end point in PRISSMM, but is less important for hematologic cancers, whereas assessment of residual disease according to blood-based assays is important for hematologic cancers but is not applicable for solid tumors. As such, the curation model would require revision and validation to appropriately account for these differences. Given the complexity of hematologic malignancies and related treatments, we anticipate that additional curator training may be needed. The other QA processes would remain largely the same, modified in terms of the addition of hematologic-specific logic checks and queries, with a potentially increased percentage of cases that undergo source data verification.

In conclusion, the GENIE BPC data contribute a standardized assessment on the basis of the PRISSMM framework of structured information from the radiology reports, pathology reports, and medical oncologist assessments, alongside important outcomes in oncology, namely treatment duration and PFS. In the setting of this pan-cancer multi-institution curation project, a comprehensive approach to assessing feasibility, accuracy, and reproducibility is necessary to ensure the highest data quality.

## Figures and Tables

**TABLE A1.**
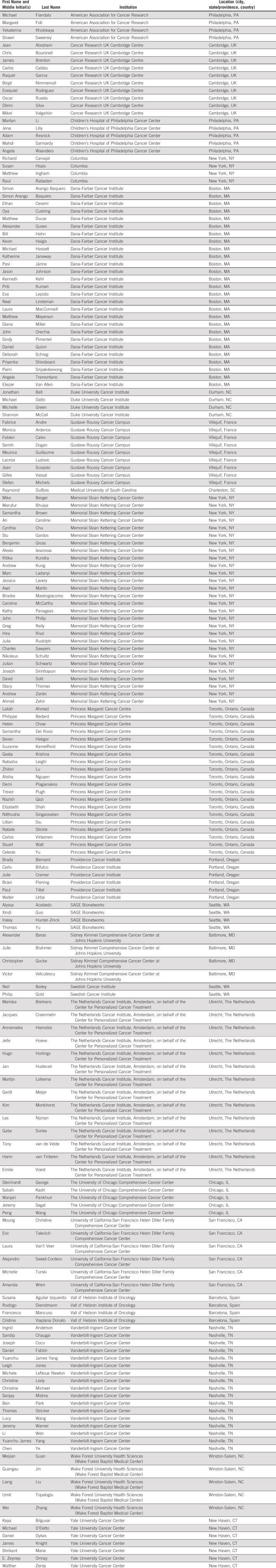
APPENDIX 1. AACR Project GENIE Consortium
